# P-1713. Blood in Bronchoalveolar Lavage Samples can Produce False-Positive Aspergillus Galactomannan Antigen Test Results

**DOI:** 10.1093/ofid/ofaf695.1885

**Published:** 2026-01-11

**Authors:** Aditi S Iyer, D J Valint, Mabel Jimenez, Steven A Pergam, Guang-Shing Cheng, David Fredricks, Denise McCulloch

**Affiliations:** Fred Hutchinson Cancer Center, Sammamish, WA; Fred Hutchinson Cancer Center, Sammamish, WA; Fred Hutch Cancer Center, Infectious Disease Division, Seattle, WA, Seattle, Washington; Fred Hutchinson Cancer Center; University of Washington, Seattle, WA; University of Washington; Fred Hutchinson Cancer Center, Seattle, Washington; Fred Hutchinson Cancer Research Center; University of Washington, Seattle, WA; Fred Hutchinson Cancer Center, Sammamish, WA

## Abstract

**Background:**

Invasive pulmonary aspergillosis (IPA) is the most common invasive fungal infection in immunocompromised patients. Bronchoalveolar lavage (BAL) fluid galactomannan assays are an important diagnostic tool, but can yield false positive results, leading to diagnostic errors and unnecessary treatment. We hypothesize that BAL fluid contaminated by blood contributes to false positive Galactomannan Optical Density Index (GM-ODI) results.

**Figure 1.**
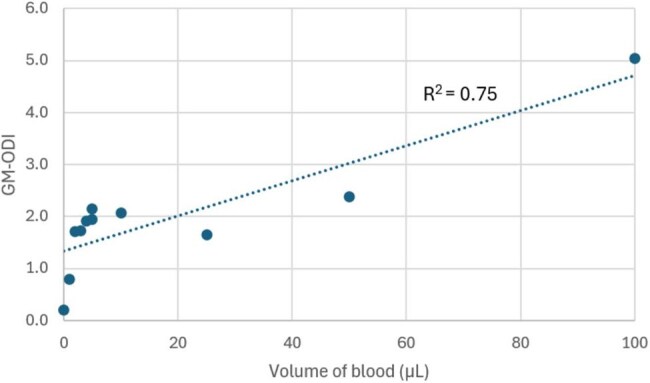
Relationship between the volume of blood added to BAL samples and the Aspergillus galactomannan optical density index (GM-ODI)

**Figure 2.**
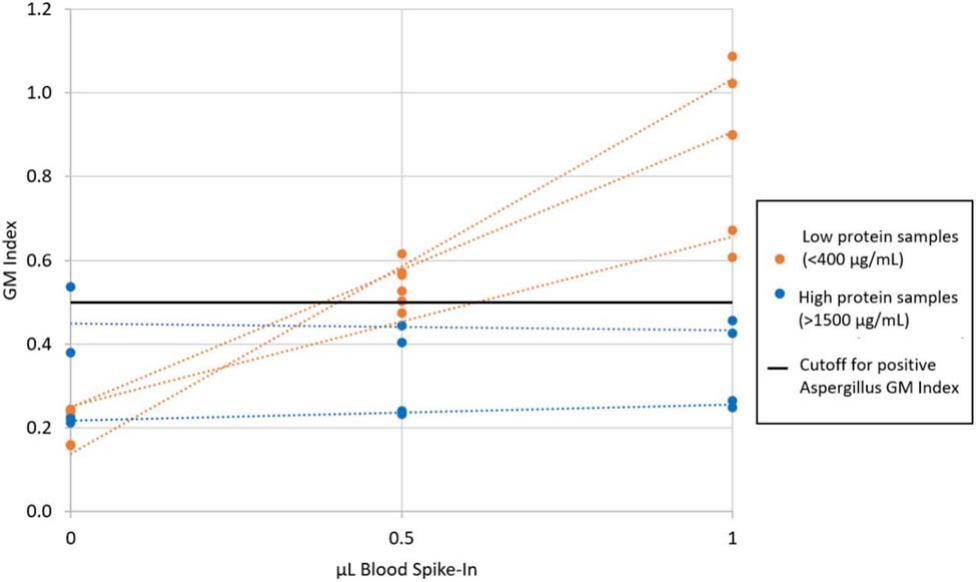
Addition of blood to GM-negative BAL samples resulted in false-positive Aspergillus GM results in low-protein samples, but no effect in high-protein samples

**Methods:**

We used leftover, frozen BAL samples from patients at Fred Hutchinson Cancer Center who underwent diagnostic bronchoscopy for pulmonary infiltrates. Samples were analyzed using the BioRad Platelia™ Aspergillus Enzyme Immunoassay. All testing included 2 technical replicates. BAL samples were spiked with incremental volumes of fresh blood (0-100 µL.) The protein concentrations of the BAL samples were measured using a Bradford Assay. Samples spanning a range of protein concentrations were spiked with 0.5 µL and 1 µL of blood to assess impact on GM-ODI. Finally, we spiked low-protein, GM-negative samples with blood (1 µL) and varying concentrations of bovine serum albumin (500 µg/mL and 1500 µg/mL) to evaluate its ability to mitigate blood's impact on the GM Index.

**Figure 3.**
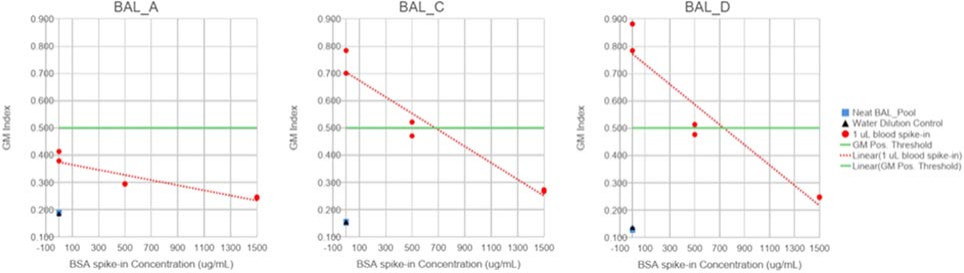
Addition of blood to GM-negative pooled BAL samples resulted in a false-positive GM that was then mitigated by the addition of bovine serum albumin

**Results:**

The addition of blood increased GM-ODI values in a dose-dependent manner (Figure 1). This effect was more pronounced in BAL samples with low-protein concentrations and was not observed in those with high-protein concentrations (Figure 2). When bovine serum albumin was added to BAL fluid to increase protein concentrations, the effect of blood on false positives diminished.

**Conclusion:**

The presence of blood significantly alters the BAL GM index, particularly in BAL samples with a low protein concentration, where it can lead to false positive results. Adding protein reduces the false positive effect, which suggests that heterogeneity seen in clinical BAL samples may be in part due to differences in protein content. For BAL samples with substantial blood, such as in patients with diffuse alveolar hemorrhage, positive GM values should be evaluated with caution.

**Disclosures:**

Steven A. Pergam, MD, MPH, F2G: Site PI for clinical trial|Global Life Technologies, Inc.: Grant/Research Support|Mundipharma: Site PI for clinical trial|Symbio: Site PI for clinical trial Guang-Shing Cheng, MD, Janssen: Grant/Research Support|Sanofi: Honoraria David Fredricks, MD, BD: Royalty|Seres Therapeutics: Advisor/Consultant Denise McCulloch, MD, MPH, Pfizer: Grant/Research Support

